# Development of an in vitro human alveolar epithelial air-liquid interface model using a small molecule inhibitor cocktail

**DOI:** 10.1186/s12860-024-00507-7

**Published:** 2024-03-18

**Authors:** Ikuya Tanabe, Kanae Ishimori, Shinkichi Ishikawa

**Affiliations:** Scientific Product Assessment Center, R&D Group, Japan Tobacco Inc., 6-2 Umegaoka, Aoba-ku, Yokohama, Kanagawa 227-8512 Japan

**Keywords:** Alveolar epithelial cells, Air-liquid interface, Y-27632, A-83-01, CHIR99021, Surfactant proteins

## Abstract

**Background:**

The alveolar epithelium is exposed to numerous stimuli, such as chemicals, viruses, and bacteria that cause a variety of pulmonary diseases through inhalation. Alveolar epithelial cells (AECs) cultured in vitro are a valuable tool for studying the impacts of these stimuli and developing therapies for associated diseases. However, maintaining the proliferative capacity of AECs in vitro is challenging. In this study, we used a cocktail of three small molecule inhibitors to cultivate AECs: Y-27632, A-83-01, and CHIR99021 (YAC). These inhibitors reportedly maintain the proliferative capacity of several types of stem/progenitor cells.

**Results:**

Primary human AECs cultured in medium containing YAC proliferated for more than 50 days (over nine passages) under submerged conditions. YAC-treated AECs were subsequently cultured at the air-liquid interface (ALI) to promote differentiation. YAC-treated AECs on ALI day 7 formed a monolayer of epithelial tissue with strong expression of the surfactant protein-encoding genes *SFTPA1*, *SFTPB*, *SFTPC*, and *SFTPD*, which are markers for type II AECs (AECIIs). Immunohistochemical analysis revealed that paraffin sections of YAC-treated AECs on ALI day 7 were mainly composed of cells expressing surfactant protein B and prosurfactant protein C.

**Conclusions:**

Our results indicate that YAC-containing medium could be useful for expansion of AECIIs, which are recognized as local stem/progenitor cells, in the alveoli.

## Background

Cellular respiration is a vital process for energy production in all living organisms. Human cells mainly carry out aerobic respiration, which involves the use of oxygen to create energy through oxidation of glucose into carbon dioxide and water. Breathing, therefore, plays a vital role in human survival by facilitating the exchange of oxygen and carbon dioxide gases in the pulmonary alveoli, which are located at the distal ends of the respiratory tract. However, inhalation of stimuli in the ambient air (e.g., chemicals, viruses, and bacteria) can also lead to respiratory diseases. Assessment of the impacts of these stimuli on the alveoli is important to explore and develop new therapeutic methods to treat the associated diseases.

Studies on the alveoli are generally conducted using animals or human cell models [1]. In recent years, in vitro studies in human cells have become increasingly common, not only because they provide advantages such as simplicity, speed, and inexpensiveness, but because they reflect changing viewpoints on the ethics of animal research [2]. Several in vitro models of human alveoli have been developed that primarily focus on the alveolar epithelium, which mainly comprises alveolar epithelial cells (AECs) of types I and II (AECIs and AECIIs, respectively) in vivo [3]. AECIs are thin, flat cells that are responsible for gas exchange, while AECIIs are cuboidal cells that secrete lung surfactant, which is essential for normal lung function [4–6]. The A549 cell line, derived from human lung adenocarcinoma, is commonly used as an in vitro model of alveolar epithelium [7]. Like most immortalized and tumor-derived cell lines, however, A549 cells exhibit limited characteristics of in vivo alveolar epithelium [8, 9]. Thus, there is a need for the development of human AEC lines that are more physiologically relevant [10].

The use of primary human AECs is another option for the development of in vitro alveolar epithelial models because they are expected to exhibit properties that closely resemble those of normal tissue. However, AECIs are terminally differentiated and lack proliferative capacity. Therefore, expansion of AECIIs, the progenitors of AECIs, is necessary for the development of an in vitro alveolar epithelial model using primary human cells. Several methods for culturing human AECIIs have been reported in the literature. For example, a combination of feeder cells and the Rho-associated protein kinase (ROCK) inhibitor Y-27632 has been reported to maintain the proliferative capacity of human AECIIs [11]. Proliferation of human AECIIs can also be maintained without feeder cells using spheroid cultures supplemented with various factors [12]. Furthermore, there are methods for inducing AECs from human pluripotent stem cells [13–17]. However, the reported methods for maintaining primary AEC proliferation are complex, while those for inducing AECs from pluripotent stem cells are also complex, in addition to being time-consuming and expensive. Therefore, a simple and universal culture method for proliferating primary human AECs should be included in the development of in vitro alveolar epithelial models.

The objective of this study was to develop a human in vitro alveolar epithelial model through the establishment of a simple culture method to maintain the proliferative capacity of human AECs. To achieve this, we utilized a cocktail of three inhibitors, Y-27632, A-83-01, and CHIR99021 (YAC), which have previously been found to be effective in maintaining the proliferation of rat hepatocytes or their progenitors [18, 19]. We hypothesized that YAC would also be useful in maintaining the proliferation of primary human AECs, especially AECIIs, which are recognized as local stem/progenitor cells in the alveoli [20]. Y-27632 is a ROCK inhibitor [21], A-83-01 is an inhibitor of transforming growth factor-β (TGF-β) signaling [22], and CHIR99021 is an activator of Wnt signaling via inhibition of glycogen synthase kinase-3 [23]. YAC has been utilized in various combinations to culture human stem cells [24], and previous studies used at least one of these inhibitors to maintain proliferation of primary human AECIIs [11, 12, 25]. We obtained primary human AECs and initially cultured them in flasks containing medium supplemented with YAC under submerged conditions, followed by culturing at the air-liquid interface (ALI). ALI culture mimics the in vivo environment of the alveolar epithelium and is known to promote the differentiation of AECs [26]. The YAC-treated, ALI-cultured AECs were then evaluated for the expression of alveolar epithelium-specific phenotypes. For comparison, we also evaluated ALI-cultured A549 cells that were prepared in accordance with previously described methods. A549 cells are conventionally used as an in vitro model of AECs [27, 28].

## Methods

### Cell culture

Human pulmonary AECs (HPAEpiCs) were purchased as AECs from ScienCell Research Laboratories (Carlsbad, CA, USA). AEC medium (AEpiCM, ScienCell Research Laboratories), which is composed of basal medium, fetal bovine serum (FBS), penicillin/streptomycin solution, and Epithelial Cell Growth Supplement (EpiCGS), was used for cultivation. The final concentration of penicillin and streptomycin were 100 units/mL and 100 µg/mL, respectively. AEpiCM was supplemented with 10 µM Y-27632, 0.5 µM A-83-01, and 3 µM CHIR99021 (all from FUJIFILM Wako Pure Chemical, Osaka, Japan). The HPAEpiCs used in this study were from three lots, each derived from a different fetus 20 weeks gestation: #27000, #28696, and #33625. A549 cells (American Type Culture Collection, Manassas, VA, USA) were cultured in Dulbecco’s modified Eagle medium (Thermo Fisher Scientific, Waltham, MA, USA) supplemented with 10% FBS (MP Biomedicals, Santa Ana, CA, USA), as described in previous studies [27, 28]. Cells were seeded in collagen type I-coated T75 flasks (Corning, Corning, NY, USA) at 0.5–1.0 × 10^6^ cells/flask, incubated at 37 °C in 5% CO_2_, and passaged when they reached 80–90% confluency. The population doubling level (PDL) of YAC-treated HPAEpiCs at each passage was calculated as follows: PDL = PDL_0_ + 3.322(logC_f_– logC_i_), where PDL_0_ is initial PDL, C_i_ is initial cell number seeded into flasks, and C_f_ is final cell yield, or the number of cells at each passage.

### ALI culture

YAC-treated HPAEpiCs and conventional A549 cells at passage 3 were used for ALI culture. The cells were seeded at 1 × 10^5^ cells/cm^2^ on the apical surface of Transwell inserts (6.5-mm diameter, 0.4-µm pores; Corning) in 24-well plates. Cells were cultivated in AEpiCM containing YAC under submerged conditions for 4–9 days until they reached confluency. The culture medium was then removed from the apical surface to establish an ALI culture, and the cells were further cultured for 7 days in AEpiCM containing YAC. The medium was changed every 2–3 days throughout the culture period.

### Measurement of tissue integrity

Transepithelial electrical resistance (TEER) values were assessed using a Millicell ERS-2 Voltohmmeter (Merck Millipore, Burlington, MA, USA) to assess tissue integrity during ALI culture. TEER (Ω × cm^2^) was calculated as follows: TEER = (resistance of tissue insert– resistance of blank insert) × surface area of the insert.

### Histological analysis

Cells on ALI day 7 were fixed with 4% paraformaldehyde (FUJIFILM Wako Pure Chemical), embedded in paraffin, sectioned using a rotary microtome RX-860 (Yamato Kohki, Saitama, Japan), deparaffinized, and subjected to hematoxylin and eosin or immunohistochemical staining. Deparaffinized sections were processed for immunohistochemical staining by incubation in the following series of solutions: (1) 10 mM citric acid buffer (LSI Medience, Tokyo, Japan) for 15–30 min at 95 °C; (2) blocking buffer (phosphate-buffered saline [PBS, Thermo Fisher Scientific] containing 1% bovine serum albumin [Merck Millipore], and 0.1% Triton X-100 [FUJIFILM Wako Pure Chemical]) for 30 min at room temperature (RT); (3) primary antibodies against cytokeratin 19 (CK19, diluted 1:500, ab52625; Abcam, Cambridge, UK), SOX2 (1:400, ab97959; Abcam), SOX9 (1:1000, ab185966; Abcam), aquaporin-5 (AQP-5, 1:200, ab92320; Abcam), surfactant protein B (SP-B, 1:200, ab40876; Abcam), or prosurfactant protein-C (proSP-C, 1:200, ab90716; Abcam) for 90 min at RT; (4) Alexa 488-conjugated secondary antibody (1:1000, ab150077; Abcam) or Alexa 555-conjugated secondary antibody (1:500, A-31572; Thermo Fisher Scientific) for 60 min at RT for single-labeling studies; anti-HT1-56 antibody (1:100, TB-29AHT1-56; Terrace Biotech, San Francisco, CA, USA) for 90 min at RT and Alexa 488-conjugated secondary antibody (1:500, ab150105; Abcam) for 60 min at RT for double staining studies; and (5) DAPI solution (1:1000, Dojindo, Kumamoto, Japan) for 15 min at RT. The sections were rinsed twice with PBS between each step. Images were acquired with an AXIO Imager or Observer (Carl Zeiss, Oberkochen, Germany).

### Immunocytochemical analysis

Cells on ALI day 7 were fixed with 4% paraformaldehyde, then incubated in the following series of solutions: blocking buffer for 60 min at RT; antibodies against E-cadherin (1:500, ab40772, Abcam), AQP-5 (1:100, ab92320, Abcam), or proSP-C, (1:200, ab90716, Abcam) for 90 min at RT; Alexa 488-conjugated secondary antibody (1:500, ab150077, Abcam) for 60 min at RT; and DAPI solution (1:1000, Dojindo) for 15 min at RT. The cells were rinsed twice with PBS between each step. Images were acquired by a confocal laser scanning microscope (LSM880, Carl Zeiss).

### Quantitative PCR

Total RNA was extracted from YAC-treated HPAEpiCs and A549 cells (passage 3) cultured under submerged and ALI conditions using an RNeasy Mini Kit (Qiagen, Hilden, Germany). The cDNA was synthesized using SuperScript IV VILO Mastermix (Thermo Fisher Scientific). Quantitative real-time PCR was performed using a 7900HT Fast Real-Time PCR system (Thermo Fisher Scientific) with the following TaqMan gene expression assay kits (Thermo Fisher Scientific): Hs00387048_m1 (*AQP5*), Hs00831305_s1 (*SFTPA1*), Hs00167036_m1 (*SFTPB*), Hs00161628_m1 (*SFTPC*), and Hs01108490_m1 (*SFTPD*). The following thermal cycling profile was applied to all samples: 2 min at 50 °C, 20 s at 95 °C followed by 40 cycles of 1 s at 95 °C and 20 s at 60 °C. Relative expression levels were calculated by the ΔΔCt method using 18 S rRNA (4319413E, Thermo Fisher Scientific) as an internal control. When cycle threshold (Ct) values were undetermined, calculations were performed with a Ct value of 40. If the Ct values were undetermined for an entire set of samples, expression of the gene was considered to be undetectable under those conditions. HPAEpiCs (lot #27000) cultured under submerged conditions (passage 1) were used as a calibrator (relative expression = 1).

### Statistical analysis

Data are expressed as mean ± standard error of three independent experiments, with six inserts (ALI culture) or a single flask (submerged culture) in each experiment. Statistical analysis was performed using GraphPad Prism 9 (GraphPad Software Inc., La Jolla, CA, USA) employing one-way analysis of variance (ANOVA) or two-way ANOVA with Dunnett’s multiple comparison test. A *p* value < 0.05 was considered to indicate statistical significance.

## Results

### Maintenance of AEC proliferation

**Fig. 1:**
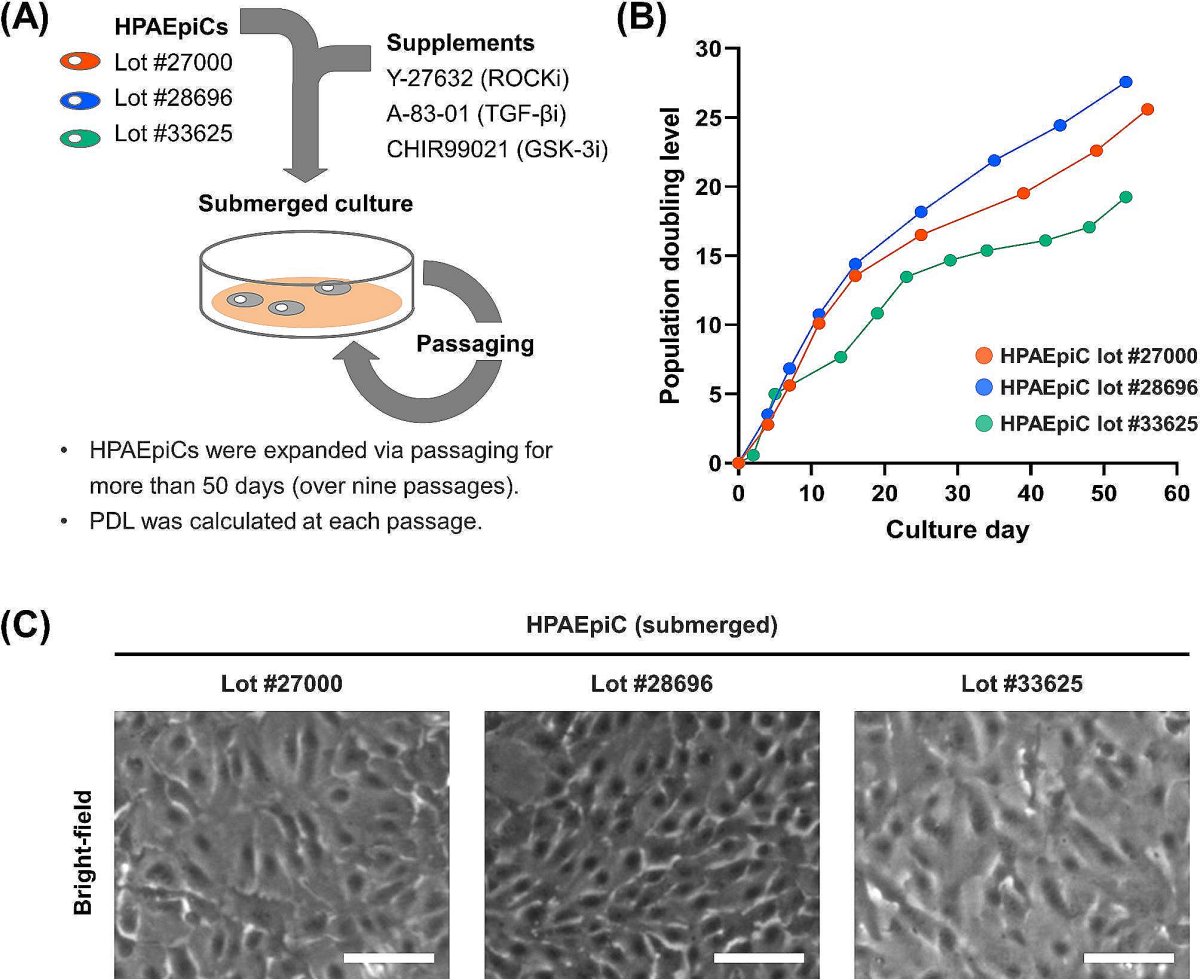
Maintenance of proliferative capacity of HPAEpiCs in AEpiCM containing YAC under submerged culture conditions. (**A**) Schematic diagram of the experimental procedure. Each plot represents the date of passaging, with over nine passages during the culture period. (**B**) Time course of PDLs of YAC-treated HPAEpiC cultures. (**C**) Phase contrast images of YAC-treated HPAEpiCs (passage 3). Scale bar: 100 μm

HPAEpiCs were purchased from ScienCell Research Laboratories as primary human AECs and cultured under submerged conditions in AEpiCM supplemented with YAC (Fig. 1A). According to the manufacturer, HPAEpiCs comprise AECIs and AECIIs and are unsuitable for expansion or long-term culture because the cells differentiate to become AECIs immediately after plating. Normally, AECIs do not proliferate in culture. We measured the PDL for more than 50 days (over nine passages) to determine whether the proliferative capacity of HPAEpiCs was maintained under treatment with YAC (Fig. 1A). Indeed, HPAEpiCs continued to proliferate throughout the entire period, regardless of the lot number (Fig. 1B). Furthermore, there were no distinct lot-to-lot differences in cell morphology at passage 3 (Fig. 1C).

### Tissue structure during ALI culture

**Fig. 2:**
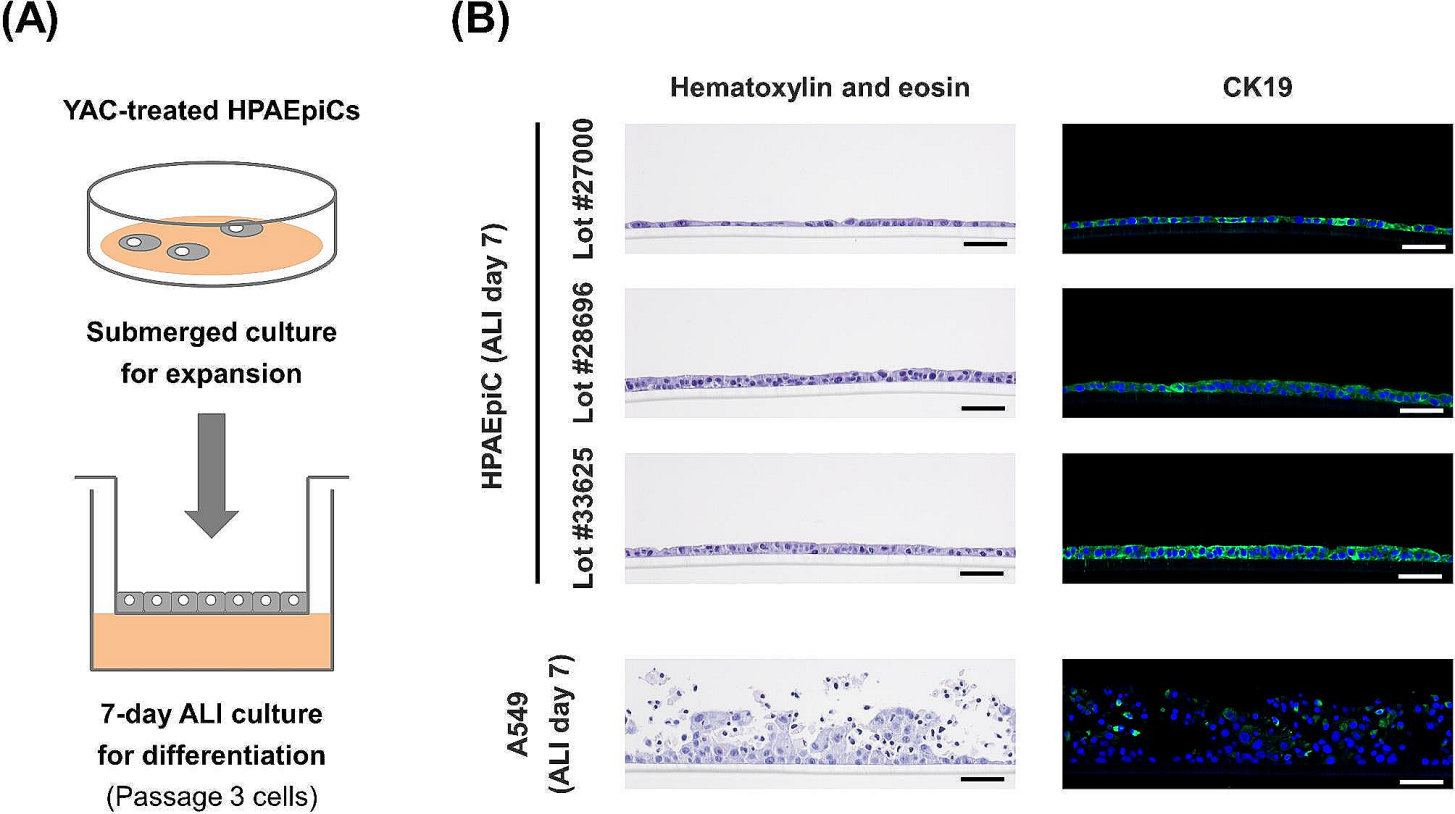
Comparative histological analysis of YAC-treated HPAEpiCs and conventional A549 cells under ALI culture conditions. (**A**) Passage 3 cells were used for 7-day ALI culture. (**B**) Representative images of hematoxylin and eosin staining (left panels) and immunohistochemical staining with anti-CK19 antibody (right panels) on day 7 of culture are shown. Blue color indicates nuclei (DAPI), and green color indicates CK19 (Alexa 488). Scale bar: 50 μm

First, we confirmed that HPAEpiCs were able to be passaged in AEpiCM containing YAC. Next, to confirm that these passaged cells had the ability to differentiate into alveolar epithelial tissue, we cultured passage 3 cells under ALI conditions for 7 days in AEpiCM containing YAC (Fig. 2A). We confirmed that YAC-treated HPAEpiCs formed monolayered tissues on the porous membrane of commercial Transwell inserts, regardless of lot number (Fig. 2B). These tissues comprised cells expressing the epithelial cell marker CK19 (Fig. 2B). By contrast, A549 cells, which are frequently used as an in vitro alveolar epithelial model, formed multilayered structures with sparse expression of CK19 when cultured at the ALI (Fig. 2B).

### Tissue integrity during ALI culture

**Fig. 3:**
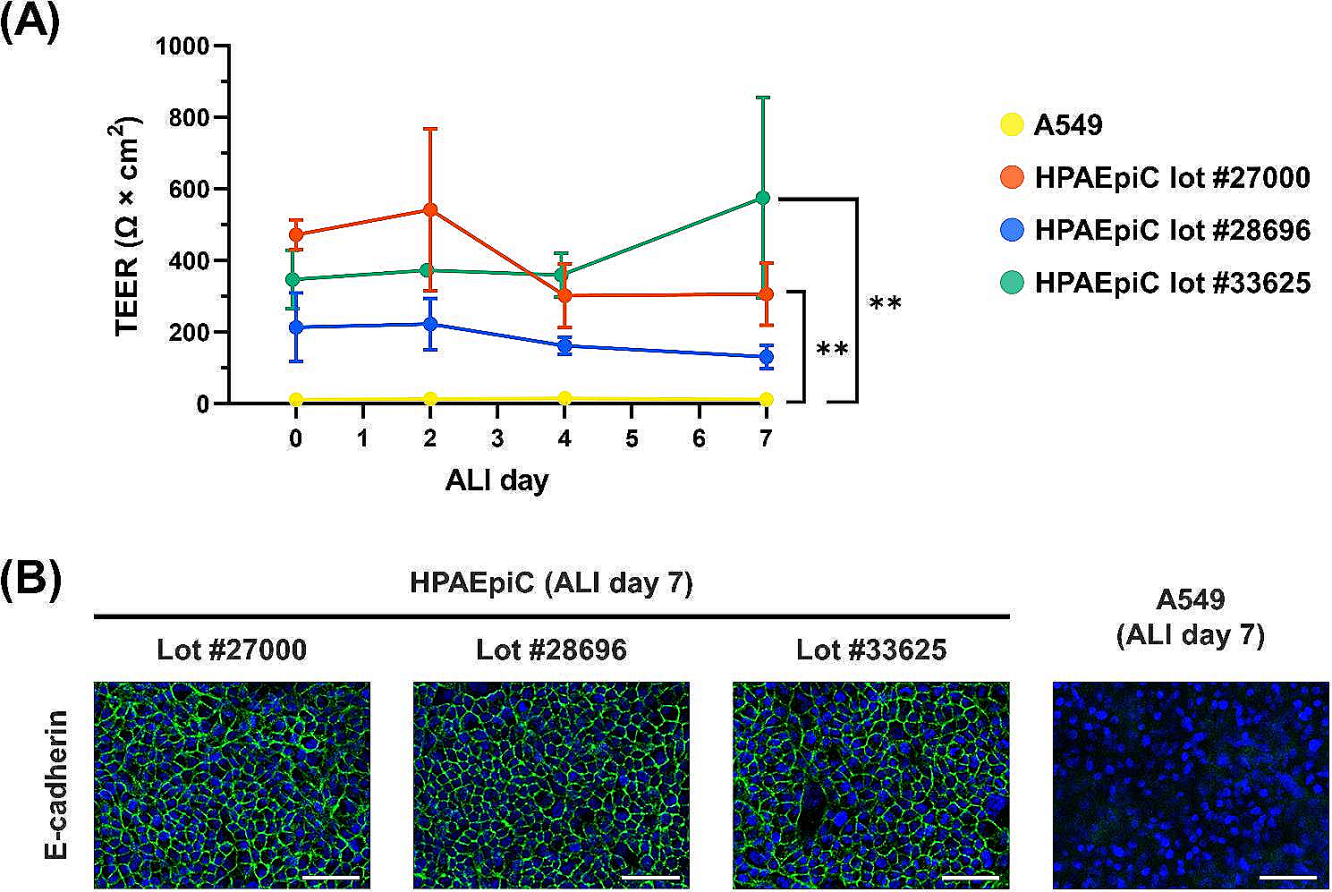
Comparative tissue integrity of YAC-treated HPAEpiCs and conventional A549 cells under ALI culture conditions. Passage 3 cells were used for ALI culture. (**A**) TEER values during ALI culture. Data expressed as mean ± standard error (*n* = 3, three independent experiments). Data obtained from each lot of HPAEpiCs were compared with those from A549 cells using two-way repeated measure ANOVA with Dunnett’s multiple comparison test; **p < 0.01. (**B**) E-cadherin expression in ALI-cultured, YAC-treated HPAEpiCs and conventional A549 cells. Blue color indicates nuclei (DAPI), and green color indicates E-cadherin (Alexa 488). Scale bar: 50 μm

Next, we assessed the integrity of epithelial tissues composed of YAC-treated HPAEpiCs formed at the ALI. TEER values, which reflect tight junction formation, exceeded 100 Ω × cm^2^ throughout the ALI culture period, regardless of the lot number (Fig. 3A). By contrast, ALI-cultured A549 cells showed very low TEER values. The epithelial tissues derived from two HPAEpiC lots (#27000 and #33625) exhibited significantly higher TEER values than those derived from ALI-cultured A549 cells (Fig. 3A). The results of immunocytochemistry revealed that the epithelial tissues derived from all three YAC-treated HPAEpiC lots expressed E-cadherin, which reflects the formation of adherens junctions, at day 7 of ALI (Fig. 3B). E-cadherin expression was sparse in ALI-cultured A549 cells (Fig. 3B).

### Investigation of alveolar epithelial marker expression

**Fig. 4:**
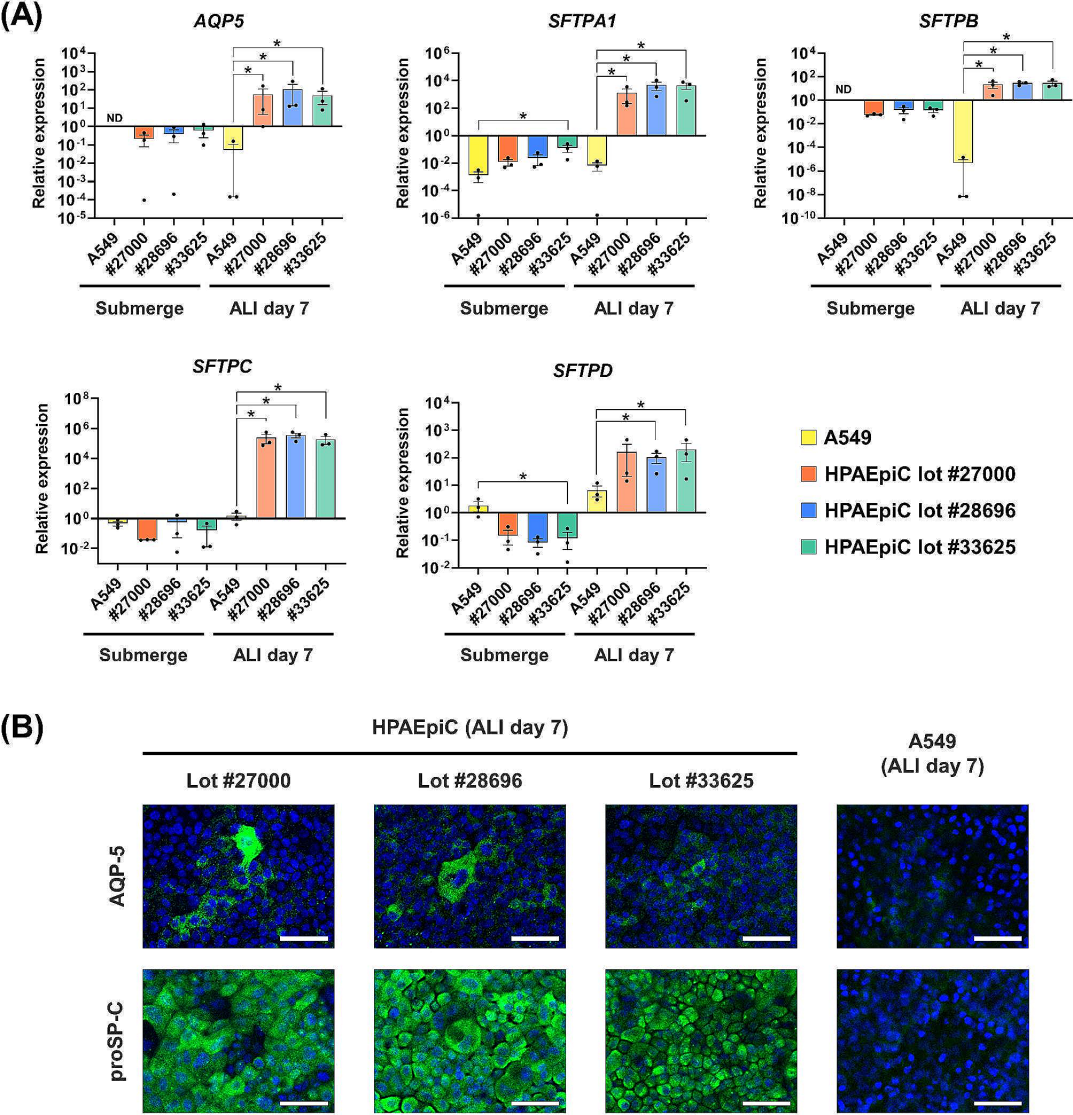
Expression of AEC markers in YAC-treated HPAEpiCs and conventional A549 cells under both submerged and ALI culture conditions. Passage 3 cells were used for analysis. (**A**) Comparative gene expression analysis of A549 cells and each lot of HPAEpiCs using *AQP5* as a marker for AECIs, and *SFTPA1*, *SFTPB*, *SFTPC*, and *SFTPD* as markers for AECIIs. Data expressed as mean ± standard error (*n* = 3, three independent experiments). Data obtained from each lot of HPAEpiCs were compared with those from A549 cells using one-way ANOVA with Dunnett’s multiple comparison test; *p < 0.05; ND, not detected. (**B**) Immunocytochemical analysis of ALI-cultured, YAC-treated HPAEpiCs and conventional A549 cells. Blue color indicates nuclei (DAPI), and green color indicates AQP-5/proSP-C (Alexa 488). Scale bar: 50 μm

Quantitative PCR was performed to compare the expression levels of alveolar epithelial marker genes in YAC-treated HPAEpiCs and conventional A549 cells cultured under submerged or ALI conditions. *AQP5* was selected as a marker for AECIs, while *SFTPA1*, *SFTPB*, *SFTPC*, and *SFTPD* were selected as markers for AECIIs. Under submerged conditions, *AQP5* and *SFTPB* expression was detected in YAC-treated HPAEpiCs, but not in A549 cells (Fig. 4A). *SFTPA1*, *SFTPC*, and *SFTPD* were expressed in both YAC-treated HPAEpiCs and conventional A549 cells under submerged conditions, but none of the genes showed significantly higher expression in YAC-treated HPAEpiCs than in A549 cells, regardless of lot number (Fig. 4A). The expression levels of all five genes were drastically increased in all three lots of YAC-treated HPAEpiCs under ALI conditions, and were significantly higher in YAC-treated HPAEpiCs than in A549 cells, except for *SFTPD* in HPAEpiC lot #27000 (Fig. 4A). Immunocytochemical analysis of YAC-treated HPAEpiCs cultured under ALI conditions revealed that AQP-5 was expressed in some cells, while proSP-C was expressed in most cells (Fig. 4B). Expression of both AQP-5 and proSP-C was weak in ALI-cultured A549 cells (Fig. 4B).

### Investigation of differentiation status of ALI-cultured HPAEpiCs

**Fig. 5:**
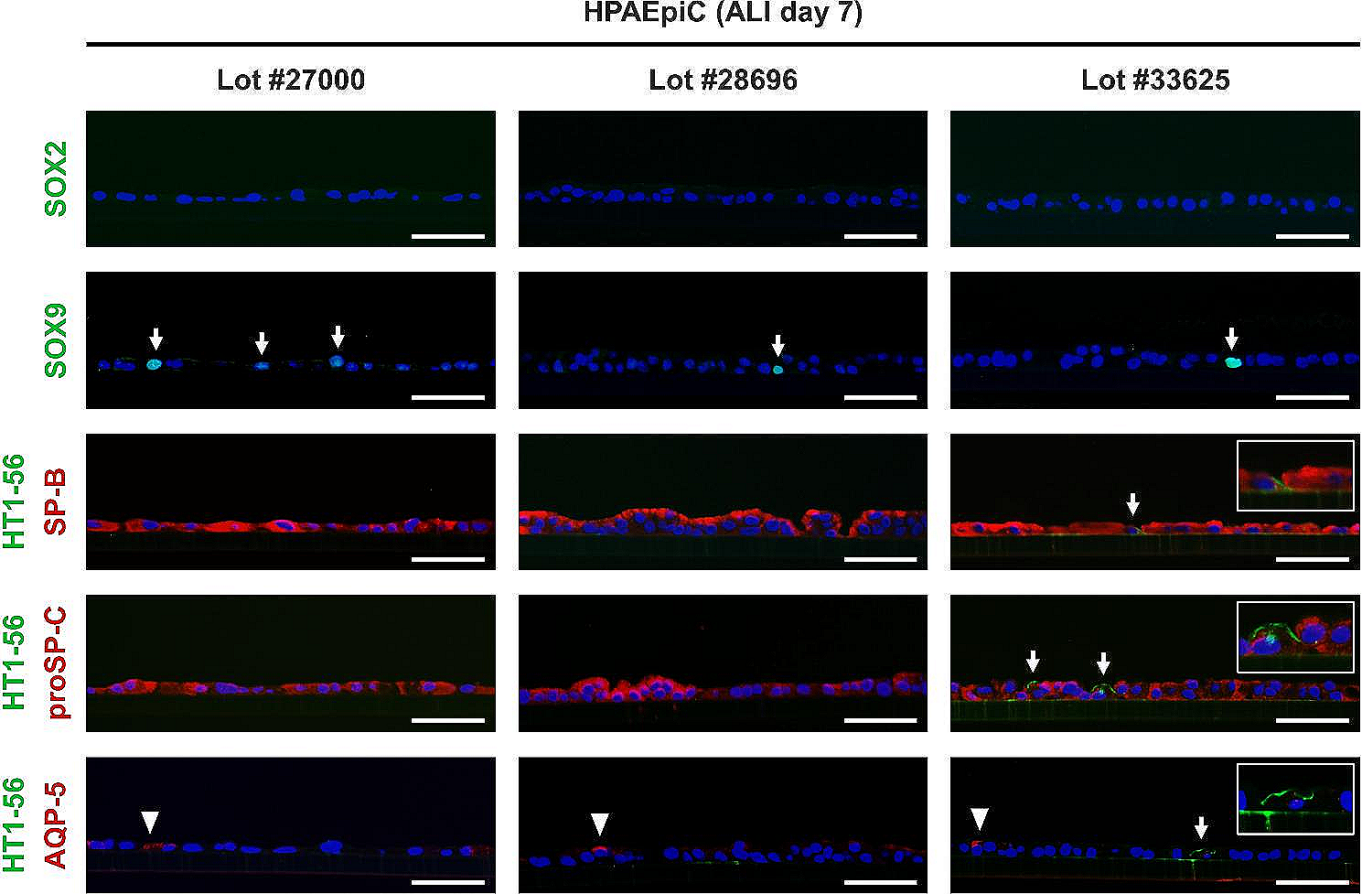
Fluorescent immunohistochemical analysis of paraffin sections obtained from YAC-treated HPAEpiCs on ALI day 7 using antibodies against markers of undifferentiated and differentiated lung epithelial cells. SOX2 and SOX9 are markers of undifferentiated progenitor cells of the proximal and distal lung bud, respectively. SOX9-positive cells are indicated by arrows. SP-B and proSP-C are markers of differentiated AECIIs. HT1-56 and AQP-5 are expressed in differentiated AECIs. HT1-56-positive cells are indicated by arrows, while AQP-5-positive cells are indicated by arrowheads. Blue color indicates nuclei (DAPI). Scale bar: 50 μm

Further characterization of the alveolar epithelial tissues composed of YAC-treated HPAEpiCs formed at the ALI was performed. The tissues were fixed on ALI day 7 and paraffin sections were prepared for immunohistochemical analysis. There were no cells with detectable expression levels of SOX2, a marker of undifferentiated proximal lung progenitor cells (Fig. 5), whereas SOX9, a marker of undifferentiated distal lung progenitor cells, was expressed in a small fraction of cells, regardless of the lot number (Fig. 5). While the expression of these undifferentiated cell markers was limited, the expression of SP-B and proSP-C, which are differentiation markers for AECIIs, was detected strongly throughout the tissue, regardless of the lot number (Fig. 5). Limited expression of HT1-56, a differentiation marker for AECIs, was observed in a very small fraction of cells in the tissue derived from only one HPAEpiC lot (#33625; Fig. 5). Furthermore, expression of these HT1-56 molecules did not co-localize with that of AQP-5, another AECI marker (Fig. 5).

## Discussion

In this study, we aimed to establish an in vitro human alveolar epithelial model for research on the pulmonary toxicity of inhaled substances and associated diseases. Using commercially available HPAEpiCs, we demonstrated that the use of YAC-containing medium maintained the proliferative capacity of the AECs for more than 50 days (over nine passages) under submerged culture, allowing their expansion. Furthermore, under ALI culture, YAC-treated HPAEpiCs formed well-organized, monolayered epithelial tissue that exhibited higher TEER values and higher expression of surfactant-encoding genes compared with A549 cells. These findings suggest that our in vitro model of ALI-cultured, YAC-treated HPAEpiCs mimics the human alveolar epithelium more closely than the traditional A549 model.

The idea that the YAC cocktail could be useful for maintaining proliferative capacity in human stem/progenitor cells was suggested by the authors of previous studies demonstrating YAC-promoted proliferation of rat hepatocytes or their progenitors [18, 29]. Therefore, we anticipated that HPAEpiC cultures grown in media supplemented with YAC might maintain the proliferative capacity of the AECIIs, which serve as local stem/progenitor cells in the alveoli. Although our YAC-treated HPAEpiCs proliferated for more than 50 days (over nine passages) under submerged culture, the details of how these small molecule inhibitors individually affect HPAEpiCs remain unclear.

Y-27632 is well known as a ROCK inhibitor [21], and is thought to reduce stress fiber formation through relaxation of the actin cytoskeleton in several cell types [30]. The possibility that this effect was involved in maintaining proliferative capacity of our AECIIs is supported by recent studies indicating that actin remodeling and cytoskeletal strain are important for terminal differentiation of AECIIs into AECIs [31, 32]. In addition to inhibiting ROCK-mediated terminal differentiation of AECIIs, Y-27632 may promote the activation of signaling pathways involved in cell proliferation and survival (e.g., Akt, ERK, and JNK) [33–35]. Y-27632 reportedly also works in combination with fibroblasts as feeder cells to maintain AECII proliferation [11], confirming its critical importance in our novel in vitro human alveolar epithelial model.

The fact that Y-27632 interacts with fibroblasts to maintain AECII proliferation suggests that A-83-01 (inhibitor of TGF-β signaling) and CHIR99021 (activator of Wnt signaling) in YAC may have substituted for the role of fibroblasts in our experiments. Lung fibroblasts have been shown to promote AECII proliferation by secreting essential soluble factors such as keratinocyte growth factor (KGF), which reportedly suppresses AECII terminal differentiation [36, 37]. KGF has also been reported to stimulate AECII proliferation [38], which can be antagonized by TGF-β [39]. Based on these reports, it is possible that A-83-01 promoted AECII proliferation in this study by blocking the antagonistic TGF-β signal. In addition to KGF, lung fibroblasts also reportedly secrete Wnt protein [40], and Wnt released from fibroblasts has been shown to act on neighboring AECIIs to control their proliferation and differentiation [41]. Therefore, CHIR99021 may have substituted for fibroblasts by supplying Wnt to the AECII niche.

Another important finding of this study was that the YAC-treated HPAEpiCs formed well-differentiated epithelial tissue at the ALI. Under submerged culture conditions, YAC-treated HPAEpiCs, but not A549 cells, expressed *AQP5* and *SFTPB*. The expression levels of other genes (*SFTPA*, *SFTPC*, and *SFTPD*) were not clearly different between YAC-treated HPAEpiCs and conventional A549 cells under submerged conditions. By contrast, at the ALI, YAC-treated HPAEpiCs expressed higher levels of all five marker genes compared with A549 cells.

In addition to gene expression, we also compared protein expression between YAC-treated HPAEpiCs and A549 cells at the ALI using immunocytochemical analysis. YAC-treated HPAEpiCs expressed AQP-5 and proSP-C more strongly than A549 cells. These markers have been previously well-characterized: AQP-5 in fully differentiated AECIs and proSP-C in fully differentiated AECIIs [42, 43].

Additional immunofluorescence analysis, using antibodies against both undifferentiated and differentiated lung cells, was performed to further characterize the differentiation status of YAC-treated HPAEpiCs at the ALI. SOX2 and SOX9 are typical markers expressed in the proximal and distal part of undifferentiated lung bud progenitor cells, respectively [44]. We found that SOX2 was not expressed in YAC-treated HPAEpiCs at the ALI, consistent with the fact that alveolar epithelial tissue is formed from distal lung buds. While SOX9 was expressed in a small fraction of cells, typical AECII markers such as SP-B and proSP-C were also strongly expressed in most of the cells. This is consistent with a previous study showing that ALI culture promotes differentiation of AECs derived from induced pluripotent stem cells, decreases expression of *SOX9*, and increases expression of *SFTP* genes [45]. Expression of the AECI marker HT1-56 was observed in a very small fraction of cells, indicating that epithelial tissue derived from HPAEpiCs mainly comprised SP-B/proSP-C-positive AECIIs. However, this HT1-56 expression was not co-localized with that of AQP-5, another marker for AECIs. One possible explanation for this is the existence of several different types of AECI-like cells that express various combinations of markers, which would include intermediate cells between AECIs and AECIIs [46]. We analyzed the expression of these markers using paraffin sections obtained from YAC-treated HPAEpiCs on ALI day 7. However, it is crucial to note that the expression patterns of these markers may undergo dynamic changes during the ALI culture. To better understand the types of cells that comprise YAC-treated HPAEpiCs and their dynamic changes during the ALI culture, we plan to perform a single-cell analysis in the future.

The observation that YAC-treated HPAEpiCs mainly comprise AECII-like cells is consistent with our hypothesis that YAC maintains the proliferation capacity of AECs, especially AECIIs. Considering that ∼ 96% of the surface area of human alveoli is covered by AECIs [47], our in vitro model may not fully replicate the physiological contributions of AECIs and AECIIs in vivo. Therefore, it will be worth investigating whether YAC-treated HPAEpiCs, which mainly comprise AECII-like cells, can differentiate into AECI-like cells. One approach for promoting the differentiation of YAC-treated HPAEpiCs into AECI-like cells is to add the Wnt signal inhibitor XAV939 to the ALI culture medium. This approach reportedly promoted the differentiation of AECIIs into AECIs [13, 25]. We anticipate that optimizing the ALI culture medium of YAC-treated HPAEpiCs will lead to ratios of AECI and AECII that more closely resemble those in vivo.

Although we successfully developed a well-differentiated alveolar epithelial model derived from human primary AECs, there are several limitations to this study. First, while we demonstrated proliferation of HPAEpiCs for more than 50 days (over nine passages), we only confirmed expression of AEC markers in YAC-treated HPAEpiCs at passage 3. Therefore, it is necessary to determine the number of passages for which the differentiation potential of YAC-treated HPAEpiCs can be maintained. Additionally, the duration of ALI culture should also be investigated further. YAC-treated HPAEpiCs were subjected to 7 days of ALI culture in this study. It would be interesting to examine the effect of longer durations of ALI culture on the differentiation status of the cells.

Second, further research is needed to determine the contribution and detailed mechanism of each YAC component in maintaining the proliferation of AECIIs in HPAEpiC. For this purpose, it will be necessary to test the effects of all combinations of the three YAC inhibitors to facilitate understanding of the detailed mechanisms of action and relative impacts of each. Third, we tested the effect of YAC on AECs using HPAEpiCs purchased from ScienCell Research Laboratories, which derived from the fetal lung. It is important to test if the effect of YAC observed in this study is also reproducible with AECs derived from the adult lung because some fetal and adult cells are known to have different characteristics.

## Conclusions

This study demonstrated that the addition of a cocktail of three small molecule inhibitors, YAC, could be used to maintain proliferative capacity of HPAEpiCs. This approach is simpler than previously reported methods using feeder cells and spheroid culture. The expanded HPAEpiCs were subjected to ALI culture to promote differentiation. YAC-treated HPAEpiCs formed a monolayered epithelial tissue, predominantly comprising the cells expressing markers for AECIIs. AECIIs are known to secrete lung surfactant to prevent alveolar collapse, and abnormalities in these cells are associated with various respiratory diseases. Therefore, this model will be useful for testing the inhalation toxicity of various substances and developing therapeutic methods for associated diseases.

## Data Availability

The datasets generated and/or analyzed during the current study are available from the corresponding author on reasonable request.

## Abbreviations

AECs: alveolar epithelial cells
AECIs: alveolar epithelial cells of types I
AECIIs: alveolar epithelial cells of types II
AEpiCM: alveolar epithelial cell medium
ALI: air-liquid interface
ANOVA: analysis of variance
AQP-5: aquaporin-5
Ct: cycle threshold
CK19: cytokeratin 19
EpiCGS: Epithelial Cell Growth Supplement
FBS: fetal bovine serum
HPAEpiCs: human pulmonary alveolar epithelial cells
PDL: population doubling level
proSP-C: prosurfactant protein-C
ROCK: Rho-associated protein kinase
RT: room temperature
TEER: transepithelial electrical resistance
TGF-β: transforming growth factor-β
YAC: Y-27632,A-83-01,and CHIR99021
